# Alterations in trimethylamine-*N*-oxide in response to Empagliflozin therapy: a secondary analysis of the EMMY trial

**DOI:** 10.1186/s12933-023-01920-6

**Published:** 2023-07-20

**Authors:** Faisal Aziz, Norbert J. Tripolt, Peter N. Pferschy, Ewald Kolesnik, Harald Mangge, Pero Curcic, Markus Hermann, Andreas Meinitzer, Dirk von Lewinski, Harald Sourij, the EMMY Investigators

**Affiliations:** 1 Interdisciplinary Metabolic Medicine Trials Unit, Graz, Austria; 2 Division of Endocrinology and Diabetology, Medical University of Graz, Graz, Austria; 3 Division of Cardiology, Medical University of Graz, Graz, Austria; 4 Clinical Institute for Medical and Chemical Laboratory Diagnostics, Medical University of Graz, Graz, Austria

**Keywords:** Clinical trial, RCT, Empagliflozin, Heart failure, Myocardial infarction, Trimethylamine *N*-oxide

## Abstract

**Introduction:**

The relationship between sodium glucose co-transporter 2 inhibitors (SGLT2i) and trimethylamine *N*-oxide (TMAO) following acute myocardial infarction (AMI) is not yet explored.

**Methods:**

In this secondary analysis of the EMMY trial (ClinicalTrials.gov registration: NCT03087773), changes in serum TMAO levels were investigated in response to 26-week Empagliflozin treatment following an AMI compared to the standard post-MI treatment. Additionally, the association of TMAO changes with clinical risk factors and cardiorenal biomarkers was assessed.

**Results:**

The mean age of patients (N = 367) was 57 ± 9 years, 82% were males, and 14% had type 2 diabetes. In the Empagliflozin group, the median TMAO value was 2.62 µmol/L (IQR: 1.81) at baseline, 3.74 µmol/L (2.81) at 6 weeks, and 4.20 µmol/L (3.14) at 26 weeks. In the placebo group, the median TMAO value was 2.90 µmol/L (2.17) at baseline, 3.23 µmol/L (1.90) at 6 weeks, and 3.35 µmol/L (2.50) at 26 weeks. The serum TMAO levels increased significantly from baseline to week 6 (coefficient: 0.233; 95% confidence interval 0.149–0.317, p < 0.001) and week 26 (0.320, 0.236–0.405, p < 0.001). The average increase in TMAO levels over time (p_interaction_ = 0.007) was significantly higher in the Empagliflozin compared to the Placebo group. Age was positively associated with TMAO, whereas eGFR and LVEF were negatively associated with TMAO.

**Conclusions:**

Our results are contrary to existing experimental studies that showed the positive impact of SGLT2i on TMAO precursors and cardiovascular events. Therefore, we recommend further research investigating the impact of SGLT2i therapy on acute and long-term changes in TMAO in cardiovascular cohorts.

**Supplementary Information:**

The online version contains supplementary material available at 10.1186/s12933-023-01920-6.

## Introduction

Trimethylamine *N*-oxide (TMAO) is a gut microbiome-derived metabolite that is synthesized from the dietary sources of choline, phosphatidylcholine, betaine, and l-carnitine [1, 2]. TMAO has recently been implicated in the development and progression of atherosclerotic cardiovascular diseases (CVD) [3]. It was shown to modulate cholesterol and lipid metabolism, promote inflammation, vascular dysfunction, and platelet hyperactivity, and facilitate the formation of macrophage foam cells and atherosclerotic plaques [4–6].

Clinical and epidemiological studies also suggest a strong association between elevated TMAO levels and adverse CVD outcomes in patients with acute cardiovascular manifestations. For example, elevated plasma TMAO levels have been associated with an increased risk of short- and long-term major adverse cardiovascular events (MACE) in patients with acute coronary syndrome and myocardial infarction [7–10]. TMAO has been correlated with a high coronary atherosclerotic burden of ST-elevation myocardial infarction (STEMI), non-ST elevation myocardial infarction (NSTEMI) [11–13], and plaque rupture in STEMI patients [14]. Our previous study showed elevated baseline TMAO levels in patients with MI-induced post-traumatic stress disorder [15]. In heart failure patients, significant correlations of TMAO with N-terminal prohormone of brain natriuretic peptide (NT-proBNP) and adverse prognosis have been documented [15, 16].

Although studies have identified TMAO as a potential risk factor and prognostic biomarker of atherosclerotic CVD, only a single measurement of TMAO has been considered in most analyses. In a large cohort study, it was shown that persistently increased TMAO levels over 10 years were associated with an increased risk of coronary heart disease and longitudinal measurements of this metabolite improved CVD risk stratification [16]. Furthermore, some studies conducted in patients with acute ischemic events have reported considerable variations in TMAO over time, which may offer a better understanding of the severity and prognosis of these events. For instance, TMAO levels declined within 24 h after myocardial infarction and increased in subsequent months, and its latter values predicted future CVD events better than its acute values [17, 18]. Another study showed that changes in TMAO levels following acute myocardial infarction (AMI) were significantly associated with MACE [19]. Similar longitudinal trajectories were observed in stroke patients at 48 h and 3 months [20]. Moreover, TMAO levels are significantly influenced by various clinical factors such as age, gender, obesity, smoking, renal function, and cardiometabolic abnormalities [1, 8, 21]. Hence, longitudinal changes in TMAO levels following the AMI with respect to these factors are worth exploring.

Sodium glucose co-transporter 2 inhibitors (SGLT2i), a class of oral glucose-lowering drugs, have been shown to significantly reduce CVD risk and improve heart failure outcomes in people with and without diabetes by targeting metabolic and anti-inflammatory mechanisms and modifying various signaling pathways [22, 23]. However, the exact mechanisms by which SGLT2i improve CVD outcomes are yet to be fully uncovered. A growing number of experimental studies have shown that SGLT2i can induce modest yet beneficial alterations in various microbiome sub-classes [24]. For instance, Empagliflozin treatment restored the gut microbiome in previously high-fat diet/streptozocin-treated mice [25]. Another study showed that SGLT2 inhibition was positively associated with TMAO precursors i.e., choline, phosphatidylcholine, and glycine [26]. While Dapagliflozin treatment caused minor alterations in the gut microbiome of diabetic mice compared to control mice [27]. These data suggest that the cardioprotective effects of SGLT2i could partly be modulated by the gut microbiome and its metabolites including TMAO. However, the direct impact of SGLT2i on TMAO and its alterations over time has not been explored.

In this study, we investigated changes in TMAO levels in response to Empagliflozin treatment initiated within 72 h following AMI for 26 weeks compared to the standard post-MI treatment. As various factors can alter TMAO levels, we also investigated its association with established clinical risk factors, cardiac function parameters, and heart failure and renal biomarkers.

## Methods

### EMMY trial

A secondary analysis of the EMMY (EMpagliflozin in acute MYocardial infarction) trial was conducted. The methodological details and primary results of the trial have been published recently [28, 29]. Briefly, the EMMY was a multicenter, randomized (1:1 ratio), double-blind, and placebo-controlled trial that investigated the effect of Empagliflozin (10 mg once daily), administered for 26 weeks, on cardiac function and heart failure biomarkers in patients with AMI.

The trial enrolled patients who underwent percutaneous coronary intervention within 72 h after AMI and were 18–80 years, haemodynamically stable, and had a blood pressure > 110/70 mmHg. Patients with other than type 2 diabetes, a blood pH < 7.32, haemodynamic instability, acute urinary tract or genital infections, on current SGLT2i therapy, or who have received SGLT2i treatment within four weeks before enrolment were excluded from the trial.

### Ethical considerations

The EMMY trial was approved by the Ethics committee of the Medical University of Graz, Austria (EK 29-179 ex16/17, EudraCT 2016-004591-22) and registered at ClinicalTrials.gov (NCT03087773). The trial conformed to the 1964 Declaration of Helsinki and adhered to the guidelines of Good Clinical Practice (ICH GCP E6).

### Outcome variable

In this secondary analysis, the outcome variable was TMAO, defined as the mean change from randomization (baseline) to 26 weeks. Fasting serum TMAO levels were measured at three timepoints: randomization, week 6, and week 26. Serum TMAO was measured with a high-performance liquid chromatography-tandem mass spectrometry method on a SCIEX QTRAP 6500 triple quadrupole instrument (Applied Biosystems, Framingham, MA, USA) [30]. Briefly, 10 µL serum was deproteinized by adding 50 µL of ice-cold methanol containing 2 µg/mL trimethylamine-13C3N-oxid (Toronto Research Chemicals, Toronto, Canada) as internal standard. After vortexing and centrifugation at 11,000*g* for 5 min, 2 μL of the supernatant was injected into the LC–MS/MS system for analysis. Separation was performed with a Hypercarb 150 × 4.6 mm, 7 µm column (Thermo) at 40 °C by isocratic elution with a mobile phase (0.01% formic acid, v/v) at a flow rate of 1.5 mL/min. TMAO and internal standards were monitored in positive multiple reaction monitoring mode using characteristic precursor–product ion transitions: m/z 76.2 → 58.2 and m/z 79.2 → 61.2, respectively. Within-day CVs for TMAO were 5.5% (2.8 µmol/L) and 2.2% (12.6 µmol/L), and between-day CVs were 9.9% (2.8 µmol/L) and 7.6% (12.6 µmol/L).

### Explanatory variables

The explanatory variables considered in this analysis were Empagliflozin versus placebo treatment groups, age, gender, smoking status, hyperlipidemia, type 2 diabetes, and measurements of body mass index (BMI), estimated glomerular filtration rate (eGFR), left-ventricular ejection fraction (LVEF), and NT-proBNP collected at baseline.

### Statistical analysis

A complete case analysis of EMMY trial participants with available TMAO measurements for all visits was performed. Continuous variables were reported as mean ± standard deviation (SD) or median with interquartile range (IQR) and categorical variables as frequencies with percentages (%). A linear mixed effect model (LMEM) was applied to analyze the change in TMAO levels over visits. The TMAO values for each visit were log-transformed using the natural logarithmic scale before adding in the LMEM and study visits, treatment, and visit-by-treatment interaction were treated as fixed effects in the model. In addition, the associations of TMAO with clinical factors (age, gender, type 2 diabetes, BMI, smoking, type of myocardial infarction, hyperlipidemia), and biomarkers (eGFR, NT-proBNP, and LVEF) were investigated using univariable and multivariable LMEMs. In the univariable LMEM, the association of TMAO over time was assessed with each risk factor and results were reported as the least square mean [LSM] ± standard error of mean (SEM) and the coefficient with corresponding 95% confidence interval (CI) and p-value. The variables found significant in the simple LMEM were simultaneously included in the multivariable LMEM along with treatment, visit, treatment-visit interaction, baseline TMAO values, age, diabetes, and sex. The results of the multivariable LMEM were reported only for significant factors. All statistical analyses were performed in the Stata software version 17.0.

## Results

The mean age of EMMY participants with available TMAO levels included in the current analysis (N = 367) at baseline was 57 ± 9 years and the majority were males (82%) and smokers (72%). Of the total participants, 14% had type 2 diabetes and 27% had hyperlipidemia. The mean BMI was 28 ± 4 kg/m^2^, eGFR was 90 ± 17 mL/min/1.73 m^2^, and LVEF was 48 ± 8%, and the median NT-proBNP was 764 pg/mL (IQR:1371). The TMAO levels and other characteristics were similar between Empagliflozin and Placebo treatment groups. See Table 1 for details.

**Table 1:** Baseline characteristics of EMMY trial participants with available TMAO measurements

Variables	All (n = 367)	Empagliflozin (n = 182)	Placebo (n = 185)	P-value
Age (years), mean ± SD	57.49 ± 9.11	57.30 ± 8.67	57.67 ± 9.57	0.700
Gender, n (%)				
Male	301 (82.02)	145 (79.67)	156 (84.32)	0.246
Female	66 (17.98)	37 (20.33)	29 (15.68)	
Smoking status, n (%)				
Smokers	263 (71.66)	130 (71.43)	133 (71.89)	0.922
No-smokers	104 (28.34)	52 (28.57)	52 (28.11)	
Hyperlipidemia, n (%)				
Yes	100 (27.25)	42 (23.08)	58 (31.35)	0.075
No	267 (72.75)	140 (76.92)	127 (86.65)	
Type 2 diabetes, n (%)				
Yes	51 (13.90)	27 (14.84)	24 (12.97)	0.606
No	316 (86.10)	155 (85.16)	161 (87.03)	
Body mass index (kg/m^2^), mean ± SD	28.11 ± 4.28	28.25 ± 4.31	27.97 ± 4.26	0.542
eGFR (mL/min/1.73 m^2^), median (IQR)	89.94 ± 17.51	90.28 ± 18.35	89.59 ± 16.68	0.709
CRP (mg/dL), median (IQR)	6.20 (12.45)	5.60 (11.20)	6.70 (13.70)	0.150
eGFR categories, n (%)				
< 60	18 (4.92)	10 (5.49)	8 (4.35)	0.724
60–90	154 (42.08)	79 (43.41)	75 (40.76)	
> 90	194 (53.01)	93 (51.10)	101 (54.89)	
LVEF (%), mean ± SD	48.21 ± 7.97	47.51 ± 7.44	48.93 ± 8.44	0.091
NT-proBNP (pg/mL), median (IQR)	764.30 (1371.00)	746.00 (1097.00)	800.45 (1623.50)	0.261
TMAO (µmol/L), median (IQR)	2.78 (1.98)	2.62 (1.81)	2.90 (2.17)	0.193

CRP: C-reactive protein, eGFR: estimated glomerular filtration rate, IQR: interquartile range, LVEF: left ventricular ejection fraction, NT-proBNP: N-terminal pro-hormone of brain natriuretic peptide, STEMI: ST elevation myocardial infarction, TMAO: trimethylamine *N*-oxide

Categorical variables were reported as frequencies and percentages (%) and compared with treatment group using Chi-square tests. Normally distributed quantitative variables were reported as mean ± standard deviation (SD) and compared with treatment group using unpaired t-tests. Non-normally distributed quantitative variables were reported as median and interquartile range (IQR) and compared with treatment group using Wilcoxon rank-sum tests

The overall median TMAO value was 2.78 µmol/L (IQR: 1.98) at baseline, 3.51 µmol/L (IQR: 2.42) at 6 weeks, and 3.71 µmol/L (IQR: 2.75) at 26 weeks. In the Empagliflozin group, the median TMAO value was 2.62 µmol/L (IQR: 1.81) at baseline, 3.74 µmol/L (IQR: 2.81) at 6 weeks, and 4.20 µmol/L (IQR: 3.14) at 26 weeks. In the placebo group, the median TMAO value was 2.90 µmol/L (IQR: 2.17) at baseline, 3.23 µmol/L (IQR: 1.90) at 6 weeks, and 3.35 µmol/L (IQR: 2.50) at 26 weeks. The IQR of TMAO increased over time in the Empagliflozin groups, whereas, it decreased in the Placebo group (Additional file 1: Fig. S1).

**Fig. 1:**
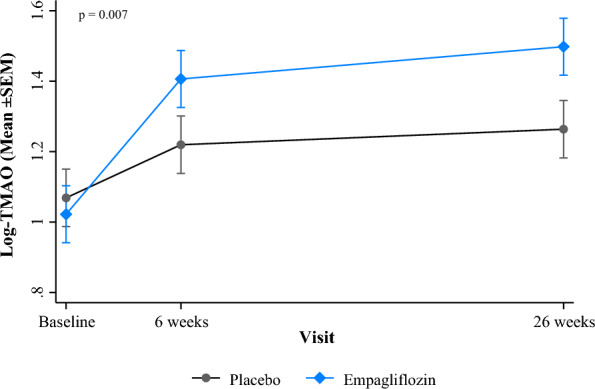
Mean ± SEM of log-TMAO levels over visits, by Empagliflozin and placebo groups

Overall TMAO levels increased significantly over time (p < 0.001 each) i.e., from baseline to week 6 (coefficient: 0.233; 95% CI 0.149–0.317, p < 0.001) and week 26 (coefficient: 0.320, 95% CI 0.236–0.405, p < 0.001) with a much more rapid increase within 6 weeks following the AMI in both groups. The mean TMAO value (coefficient: 0.117; 95% CI 0.047–0.186, p = 0.001) was significantly higher in participants on the Empagliflozin therapy than those on the Placebo over the entire follow-up period as well as at 6 weeks (p = 0.002) and 26 weeks (p < 0.001) each. The average increase in TMAO levels over time (p_interaction_ = 0.007) was also significantly higher in the Empagliflozin compared to the Placebo group (Fig. 1 and Table 1). The results remained significant after adjusting for baseline TMAO values, established CVD risk factors, and cardiorenal biomarkers.

In both unadjusted and adjusted LMEM analyses, age was positively associated with higher TMAO levels, whereas average increases in eGFR and LVEF were associated with lower TMAO levels. Other variables such as gender, BMI, smoking, type 2 diabetes and its interaction with treatment (p-interaction = 0.841), hyperlipidemia, and NT-proBNP were not associated with TMAO alterations over time (Tables 2 and 3).

**Table 2:** Average marginal means and univariable linear mixed effects regression of log-transformed TMAO levels with Empagliflozin versus placebo and other baseline variables

Variables	LSM ± SEM	Coefficient ± SEM	P-value
Visit			
Baseline	1.05 ± 0.03	Reference	
6 weeks	1.31 ± 0.03	0.268 ± 0.042	< 0.001
26 weeks	1.38 ± 0.03	0.336 ± 0.042	< 0.001
Treatment group			
Placebo	1.18 ± 0.02	Reference	
Empagliflozin	1.31 ± 0.02	0.123 ± 0.034	0.001
Gender			
Female	1.21 ± 0.04	Reference	
Male	1.25 ± 0.02	0.044 ± 0.045	0.319
Age—5 years change	–	0.046 ± 0.009	< 0.001
BMI—kg/m^2^	–	− 0.003 ± 0.004	0.427
Smoking status			
Non-smoker	1.31 ± 0.03	Reference	
Smoker	1.22 ± 0.02	− 0.086 ± 0.038	0.024
Type 2 diabetes			
No	1.24 ± 0.02	Reference	
Yes	1.31 ± 0.05	0.074 ± 0.049	0.137
Hyperlipidemia			
No	1.24 ± 0.02	Reference	
Yes	1.27 ± 0.03	0.026 ± 0.038	0.504
Type of MI			
NSTEMI	1.22 ± 0.02	Reference	
STEMI	1.25 ± 0.05	0.026 ± 0.050	0.604
eGFR—10 mL/min/1.73 m^2^ change	–	− 0.037 ± 0.010	< 0.001
eGFR categories			
< 60	1.39 ± 0.08	Reference	
60–90	1.30 ± 0.03	− 0.084 ± 0.081	0.300
> 90	1.19 ± 0.02	− 0.194 ± 0.081	0.017
LVEF—5% change	–	− 0.022 ± 0.011	0.039
NT-proBNP, pg/mL	–	0.018 ± 0.013	0.162

BMI: body mass index, eGFR: estimated glomerular filtration rate, LSM: least square mean, LVEF: left ventricular ejection fraction, NT-proBNP: N-terminal pro-hormone of brain natriuretic peptide, SEM: standard error of mean, STEMI: ST-segment elevation myocardial infarction, NSTEMI: non-ST-segment elevation myocardial infarction

**Table 3:** Multiple mixed linear effect regression of log-transformed TMAO levels with Empagliflozin versus placebo and other variables

Variables	Coefficient ± SEM	P-value
Visit		
Baseline	Reference	
6 weeks	0.254 ± 0.043	0.041
26 weeks	0.326 ± 0.043	0.005
Treatment group		
Placebo	Reference	
Empagliflozin	0.117 ± 0.035	0.001
Age—5 years change	0.023 ± 0.011	0.042
eGFR—10 mL/min/1.73 m^2^ change	− 0.025 ± 0.011	0.023
LVEF—5% change	− 0.023 ± 0.011	0.035

eGFR: estimated glomerular filtration rate, LVEF: left ventricular ejection fraction, SEM: standard error of mean

## Discussion

We investigated changes in plasma TMAO levels in patients who underwent PCI following an AMI and were randomized to the Empagliflozin therapy for 26 weeks compared to those on placebo. In addition, we ascertained the association of TMAO changes with various baseline clinical risk factors and cardiorenal biomarkers. Serum TMAO levels increased over time following MI in both treatment groups; however, the average increase in TMAO levels over time was substantially higher in the Empagliflozin versus the placebo group. Among the investigated clinical factors and biomarkers, age was positively associated with TMAO, while LVEF and eGFR were negatively associated with TMAO.

Our data revealed an increase in TMAO levels from baseline (less than 72 h after AMI) to 6 weeks and 26 weeks in both Empagliflozin and placebo patients. This persistent rise in TMAO levels could reflect an overactive inflammatory response, disturbances in metabolism, and imbalance in gut microbiome following an AMI, particularly within the first 6 weeks, and then stabilizes to some extent over the course of time, and hence may provide valuable predictive and prognostic information in this patient population. However, we did not assess the association of longitudinal values of TMAO with subsequent CVD outcomes, as the study duration was only 26 weeks. A study from Japan also showed a significant rise (p = 0.048) in TMAO values from the acute (5.63 μM) to the chronic (6.76 μM) phase of STEMI (10 months). In addition, chronic phase TMAO significantly predicted future CVD events, but not acute phase TMAO [18]. Two studies measured TMAO at admission and 1-month follow-up in patients with AMI and found that patients with high TMAO values at both timepoints showed a higher risk of MACE, whereas patients with high TMAO levels at admission and low levels at follow-up showed no association with MACE risk [19, 31]. Similarly, a decline in TMAO levels from admission (4.13 ± 4.37 μM) to 24 h (3.41 ± 5.84 μM), and an increase from 24 h to 4 months (3.70 ± 3.86 μM) in STEMI patients was reported by another recent study. Also, a decline in TMAO from admission to 4 months was inversely correlated (rho = − 0.16, p = 0.024) with infarct size [17]. The results of these studies underscore the clinical importance of repeated measurements of TMAO and its different fluctuation patterns during acute and chronic phases of post-ischemic events for predicting future CVD events.

Interestingly, the post-MI rise in TMAO levels at each visit in our study was lower than the levels reported in previous study cohorts from the Netherlands, Japan, and China [17–19]. This finding is intriguing and raises the question whether this modest rise in TMAO is clinically significant for increasing the risk of future CVD events. Also, previous studies have shown that TMAO levels fell considerably after AMI and stroke [17, 20] and then returned to normal, which could also be the same for our data; however, we could not confirm it due to the unavailability of TMAO data before the MI. Moreover, a recent mass spectrometric analysis of 694 individuals without clinical CVD, CKD, and type 2 diabetes revealed that the median level of TMAO was 3.91 μmol/L (IQR: 2.87–6.10) in men and 3.56 μmol/L (IQR: 2.41–5.15) in women [32]. Another mass spectrometric analysis showed a median TMAO concentration of 3.45 μM (2.25–5.79) in healthy individuals [33]. Hence, TMAO levels in our post-MI population are not significantly different from the general population and its rise over time may reflect the normalization of the gut microbiome after the acute ischemic event. It is important to note that the quantitative comparison of TMAO levels across studies is challenging because previous studies have shown vast differences in TMAO levels in both different and same CVD cohorts due to differences in TMAO quantification standards and methods [6, 34, 35].

Our analysis revealed that TMAO levels increased at a higher rate during 26-week follow-up in patients receiving Empagliflozin therapy compared to the placebo group after AMI with a higher dispersion over visits in the former group. It is a surprising finding, as we expected a decline or at least a lower increase in TMAO in the Empagliflozin group than placebo. This expectation was based on the assumption that the SGLT2i have well-known cardioprotective effects, which could also be exerted by dysbiosis of microbiome colonies resulting from glucose absorption impairment in the small intestine and glucose delivery facilitation in the large intestine. Such changes in the gut microbiome have been shown to significantly alter the synthesis and regulation of various metabolites including TMAO precursors such as choline, phosphatidylcholine, betaine, and l-carnitine, as demonstrated by experimental studies [24–26]. Clinical studies also support that high levels of TMAO predict worse MACE outcomes and poor CVD prognosis [1, 18]. As SGLT2i are known to reduce the eGFR shortly after the initiation of the treatment [36], a non-significant dip was also observed in our study. However, eGFR levels were similar in both groups after 26 weeks of treatment; hence renal function cannot explain our finding on both increase in TMAO levels and its variability over time in patients receiving Empagliflozin compared to placebo. In addition, how SGLT2i interfere with TMAO secretion or filtration is also not well known. The increase in TMAO variability over visits in the Empagliflozin group can be explained by some extreme TMAO values in this group in our trial population. However, TMAO is a sensitive metabolite and the data from the general population and CVD cohorts demonstrate that its distribution is highly skewed could be influenced by a vast number of demographic, clinical, dietary, and biological factors, and the concomitant pharmacotherapy, particularly in the post-hospitalization period [1, 32, 33]. Nevertheless, the EMMY study being a multi-site randomized clinical trial minimizes the impact of such factors on the observed pattern of TMAO alterations in response to Empagliflozin, as these were well-balanced in both treatment groups. We also adjusted for various known factors in our analysis to account for their residual confounding. The BIOSTAT-CHF study also supports this claim, as it reported no significant impact of standard heart failure therapy on TMAO levels [31]. Notwithstanding, the evidence on the exact mechanisms by which SGLT2i can influence TMAO concentrations in post-MI cohorts is still premature.

We investigated the association of TMAO with various factors and biomarkers such as age, smoking, BMI, hyperlipidemia, diabetes, type of myocardial infarction, eGFR, LVEF, and NT-proBNP. In our analysis, LVEF and eGFR were found to be inversely associated with increasing TMAO levels. The link between TMAO and kidney function has been increasingly recognized as plasma TMAO is excreted by kidneys, and its levels have been shown to increase with the degree of renal impairment in healthy individuals and those with heart failure and chronic kidney disease [32, 37, 38]. Also, enhanced uraemia in kidney disease could elevate TMAO levels by unsettling the gut microbiome [39]. The relationship between cardiac function and TMAO has been investigated in various CVD cohorts with contrasting results. Interestingly, it was found to be associated with advanced left ventricular diastolic dysfunction, but not with systolic dysfunction in patients with chronic heart failure [14]. In heart failure mice models, TMAO was significantly associated with worse LVEF [40]. In contrast, dietary supplementation of TMAO drastically reduced diastolic dysfunction in heart failure mice models [41]. Whereas TMAO was not associated with left ventricular systolic dysfunction at 30-day follow-up in patients with STEMI [42]. These conflicting results in animal and clinical studies accentuate the need for more longitudinal studies on TMAO and various cardiac function parameters in CVD cohorts and the impact of cardiovascular disease modifying drugs on TMAO levels and their predictive potential.

## Limitations

This study is not without limitations. Post-MI trajectories of TMAO are greatly influenced by renal function, as it is mainly excreted through urine. The EMMY trial excluded patients with eGFR below 45 mL/min/1.73 m^2^, and the mean eGFR of study participants was above the normal range (92 mL/min/1.73 m^2^). Therefore, the degree of association between TMAO and eGFR in our data might be attenuated due to this exclusion criterion. Moreover, dietary intake is well-known to impact TMAO levels considerably [43], whereas dietary information was not prospectively collected in the EMMY trial. Last, we did not measure trimethylamine, the precursor metabolised by hepatic flavin monooxygenases 3 to TMAO. Hence, whether Empagliflozin potentially affects TMA generation or metabolisation to TMAO remains to be further elucidated.

## Conclusions

To conclude, TMAO levels increased significantly over time following myocardial infarction, with a greater increase in people receiving Empagliflozin therapy compared to those on placebo. These results are surprising and in contrast with existing experimental data that demonstrated the beneficial impact of SGLT2i on the gut microbiome and TMAO precursors and an increased risk of subsequent MACE in association with sustained high TMAO levels. In light of these findings, we recommend more studies investigating acute and long-term alterations in TMAO in response to SGLT2i therapy and their impact on future CVD outcomes and prognosis.

## Supplementary Information

**Additional file 1: Fig. S1. **Distribution of untransformed TMAO concentration (μmol/L) by treatment groups at A—baseline, B—6 weeks, and C—26 weeks. **Table S1. **LDL-C levels over visits by treatment groups.

## Data Availability

Researchers can access the data upon reasonable request to the corresponding authors.

## Abbreviations

AMI: Acute myocardial infarction
CI: Confidence interval
CVD: Cardiovascular disease
EMMY: EMpagliflozin in acute MYocardial infarction
TMAO: Trimethylamine *N*-oxide
MACE: Major adverse cardiovascular events
NT-proBNP: N-terminal prohormone of brain natriuretic peptide
NSTEMI: Non-ST elevation myocardial infarction
SGLTi: Sodium glucose co-transporter 2 inhibitor
STEMI: ST elevation myocardial infarction

## References

[1] Al-Rubaye H, Perfetti G, Kaski J-C (2019). The role of microbiota in cardiovascular risk: focus on trimethylamine oxide. Curr Probl Cardiol.

[2] Gatarek P, Kaluzna-Czaplinska J (2021). Trimethylamine N-oxide (TMAO) in human health. EXCLI J.

[3] Li D, Lu Y, Yuan S, Cai X, He Y, Chen J (2022). Gut microbiota-derived metabolite trimethylamine-N-oxide and multiple health outcomes: an umbrella review and updated meta-analysis. Am J Clin Nutr.

[4] Xu H, Wang X, Feng W, Liu Q, Zhou S, Liu Q (2020). The gut microbiota and its interactions with cardiovascular disease. Micriob Biotechnol.

[5] Gong D, Zhang L, Zhang Y, Wang F, Zhao Z, Zhou X (2019). Gut microbial metabolite trimethylamine N-oxide is related to thrombus formation in atrial fibrillation patients. Am J Med Sci.

[6] Li XS, Obeid S, Klingenberg R, Gencer B, Mach F, Räber L (2017). Gut microbiota-dependent trimethylamine N-oxide in acute coronary syndromes: a prognostic marker for incident cardiovascular events beyond traditional risk factors. Eur Heart J.

[7] Waleed KB, Tse G, Lu Y-K, Peng C-N, Tu H, Ding L-G (2021). Trimethylamine N-oxide is associated with coronary atherosclerotic burden in non-ST-segment myocardial infarction patients: SZ-NSTEMI prospective cohort study. Rev Cardiovasc Med.

[8] Zhou X, Jin M, Liu L, Yu Z, Lu X, Zhang H (2020). Trimethylamine N-oxide and cardiovascular outcomes in patients with chronic heart failure after myocardial infarction. ESC Heart Fail.

[9] Li N, Zhou J, Wang Y, Chen R, Li J, Zhao X (2022). Association between trimethylamine N-oxide and prognosis of patients with acute myocardial infarction and heart failure. ESC Heart Fail.

[10] Senthong V, Wang Z, Li XS, Fan Y, Wu Y, Wilson Tang WH (2016). Intestinal microbiota-generated metabolite trimethylamine-N-oxide and 5-year mortality risk in stable coronary artery disease: the contributory role of intestinal microbiota in a COURAGE-like patient cohort. J Am Heart Assoc.

[11] Sheng Z, Tan Y, Liu C, Zhou P, Li J, Zhou J (2019). Relation of circulating trimethylamine N-oxide with coronary atherosclerotic burden in patients with ST-segment elevation myocardial infarction. Am J Cardiol.

[12] Tan Y, Sheng Z, Zhou P, Liu C, Zhao H, Song L (2019). Plasma trimethylamine N-oxide as a novel biomarker for plaque rupture in patients with ST-segment-elevation myocardial infarction. Circ Cardiovasc Interv.

[13] Baranyi A, Enko D, von Lewinski D, Rothenhäusler H-B, Amouzadeh-Ghadikolai O, Harpf H (2021). Assessment of trimethylamine N-oxide (TMAO) as a potential biomarker of severe stress in patients vulnerable to posttraumatic stress disorder (PTSD) after acute myocardial infarction. Eur J Psychotraumatol.

[14] Tang WHW, Wang Z, Shrestha K, Borowski AG, Wu Y, Troughton RW (2015). Intestinal microbiota-dependent phosphatidylcholine metabolites, diastolic dysfunction, and adverse clinical outcomes in chronic systolic heart failure. J Card Fail.

[15] Dong Z, Zheng S, Shen Z, Luo Y, Hai X (2021). Trimethylamine N-oxide is associated with heart failure risk in patients with preserved ejection fraction. Lab Med.

[16] Heianza Y, Ma W, DiDonato JA, Sun Q, Rimm EB, Hu FB (2020). Long-term changes in gut microbial metabolite trimethylamine N-oxide and coronary heart disease risk. JACC.

[17] Almesned MA, Prins FM, Lipšic E, Connelly MA, Garcia E, Dullaart RPF (2021). Temporal course of plasma trimethylamine N-oxide (TMAO) levels in ST-elevation myocardial infarction. J Clin Med.

[18] Matsuzawa Y, Nakahashi H, Konishi M, Sato R, Kawashima C, Kikuchi S (2019). Microbiota-derived trimethylamine N-oxide predicts cardiovascular risk after STEMI. Sci Rep.

[19] Li N, Wang Y, Zhou J, Chen R, Li J, Zhao X (2022). Association between the changes in trimethylamine N-oxide-related metabolites and prognosis of patients with acute myocardial infarction: a prospective study. J Cardiovasc Dev Dis.

[20] Schneider C, Okun JG, Schwarz KV, Hauke J, Zorn M, Nürnberg C (2020). Trimethylamine-N-oxide is elevated in the acute phase after ischaemic stroke and decreases within the first days. Eur J Neurol.

[21] Ringel C, Dittrich J, Gaudl A, Schellong P, Beuchel CF, Baber R (2021). Association of plasma trimethylamine N-oxide levels with atherosclerotic cardiovascular disease and factors of the metabolic syndrome. Atherosclerosis.

[22] Wang A, Li Z, Zhuo S, Gao F, Zhang H, Zhang Z (2022). Mechanisms of cardiorenal protection with SGLT2 inhibitors in patients with T2DM based on network pharmacology. Front Cardiovasc Med.

[23] von Lewinski D, Benedikt M, Tripolt N, Wallner M, Sourij H, Kolesnik E (2021). Can sodium-glucose cotransporter 2 inhibitors be beneficial in patients with acute myocardial infarction?. Kardiol Pol.

[24] Wang D, Liu J, Zhou L, Zhang Q, Li M, Xiao X (2022). Effects of oral glucose-lowering agents on gut microbiota and microbial metabolites. Front Endocrinol.

[25] Deng L, Yang Y, Xu G (2022). Empagliflozin ameliorates type 2 diabetes mellitus-related diabetic nephropathy via altering the gut microbiota. Biochim Biophys Acta Mol Cell Biol Lipids.

[26] Xu M, Zheng J, Hou T, Lin H, Wang T, Wang S (2022). SGLT2 inhibition, choline metabolites, and cardiometabolic diseases: a mediation Mendelian randomization study. Diabetes Care.

[27] Lee DM, Battson ML, Jarrell DK, Hou S, Ecton KE, Weir TL (2018). SGLT2 inhibition via dapagliflozin improves generalized vascular dysfunction and alters the gut microbiota in type 2 diabetic mice. Cardiovasc Diabetol.

[28] Tripolt NJ, Kolesnik E, Pferschy PN, Verheyen N, Ablasser K, Sailer S (2020). Impact of EMpagliflozin on cardiac function and biomarkers of heart failure in patients with acute MYocardial infarction-The EMMY trial. Am Heart J.

[29] von Lewinski D, Kolesnik E, Tripolt NJ, Pferschy PN, Benedikt M, Wallner M (2022). Empagliflozin in acute myocardial infarction: the EMMY trial. Eur Heart J.

[30] Enko D, Zelzer S, Baranyi A, Herrmann M, Meinitzer A (2020). Determination of trimethylamine-N-oxide by a simple isocratic high-throughput liquid-chromatography tandem mass-spectrometry method. Clin Lab.

[31] Suzuki T, Yazaki Y, Voors AA, Jones DJL, Chan DCS, Anker SD (2019). Association with outcomes and response to treatment of trimethylamine N-oxide in heart failure: results from BIOSTAT-CHF. Eur J Heart Fail.

[32] Gessner A, di Giuseppe R, Koch M, Fromm MF, Lieb W, Maas R (2020). Trimethylamine-N-oxide (TMAO) determined by LC-MS/MS: distribution and correlates in the population-based PopGen cohort. Clin Chem Lab Med.

[33] Wang Z, Levison BS, Hazen JE, Donahue L, Li X-M, Hazen SL (2014). Measurement of trimethylamine-N-oxide by stable isotope dilution liquid chromatography tandem mass spectrometry. Anal Biochem.

[34] Schiattarella GG, Sannino A, Toscano E, Giugliano G, Gargiulo G, Franzone A (2017). Gut microbe-generated metabolite trimethylamine-N-oxide as cardiovascular risk biomarker: a systematic review and dose-response meta-analysis. Eur Heart J.

[35] Heianza Y, Ma W, Manson JE, Rexrode KM, Qi L (2017). Gut microbiota metabolites and risk of major adverse cardiovascular disease events and death: a systematic review and meta-analysis of prospective studies. J Am Heart Assoc.

[36] Heerspink HJL, Cherney DZI (2021). Clinical implications of an acute dip in eGFR after SGLT2 inhibitor initiation. Clin J Am Soc Nephrol.

[37] Missailidis C, Hällqvist J, Qureshi AR, Barany P, Heimbürger O, Lindholm B (2016). Serum trimethylamine-N-oxide is strongly related to renal function and predicts outcome in chronic kidney disease. PLoS ONE.

[38] Guo F, Qiu X, Tan Z, Li Z, Ouyang D (2020). Plasma trimethylamine n-oxide is associated with renal function in patients with heart failure with preserved ejection fraction. BMC Cardiovasc Disord.

[39] Ramezani A, Raj DS (2014). The gut microbiome, kidney disease, and targeted interventions. J Am Soc Nephrol.

[40] Organ CL, Otsuka H, Bhushan S, Wang Z, Bradley J, Trivedi R (2016). Choline diet and its gut microbe-derived metabolite, trimethylamine N-Oxide, exacerbate pressure overload-induced heart failure. Circ Heart Fail.

[41] Huc T, Drapala A, Gawrys M, Konop M, Bielinska K, Zaorska E (2018). Chronic, low-dose TMAO treatment reduces diastolic dysfunction and heart fibrosis in hypertensive rats. Am J Physiol Heart Circ Physiol.

[42] Zhou J, Yu S, Tan Y, Zhou P, Liu C, Sheng Z (2020). Trimethylamine N-oxide was not associated with 30-day left ventricular systolic dysfunction in patients with a first anterior ST-segment elevation myocardial infarction after primary revascularization: a sub-analysis from an optical coherence tomography registry. Front Cardiovasc Med.

[43] Koay YC, Chen Y-C, Wali JA, Luk AWS, Li M, Doma H (2021). Plasma levels of trimethylamine-N-oxide can be increased with “healthy” and “unhealthy” diets and do not correlate with the extent of atherosclerosis but with plaque instability. Cardiovasc Res.

