# Supplementary material for: Alterations in trimethylamine-N-oxide in response to Empagliflozin therapy: a secondary analysis of the EMMY trial
Source: Cardiovasc Diabetol. 2023 Jul 20;22:184. doi: 10.1186/s12933-023-01920-6 (PMC10357596; doi:10.1186/s12933-023-01920-6)
Supplement: Supplementary file 1 — Additional file 1: Fig. S1. Distribution of untransformed TMAO concentration (μmol/L) by treatment groups at A—baseline, B—6 weeks, and C—26 weeks. Table S1. LDL-C levels over visits by treatment groups. [file 12933_2023_1920_MOESM1_ESM.docx]

**Additional file 1**

**Fig. S1.** Distribution of untransformed TMAO concentration (µmol/L) by treatment groups at A–baseline, B–6 weeks, and C–26 weeks.

IQR: Interquartile range

**Table S1.** LDL-C levels over visits by treatment groups.

| LDL-C | Visit 1 | 6 weeks | 26 weeks | P-value | P-interaction |
| --- | --- | --- | --- | --- | --- |
| Empagliflozin | 123.2 ±37.9 | 62.7 ±26.4 | 61.0 ±28.6 | <0.001 | 0.579 |
| Placebo | 122.6 ±42.5 | 60.3 ±25.3 | 56.0 ±23.8 | <0.001 |  |
| LDL-C: Low density lipoprotein cholesterol  P-values are derived from the linear mixed | | | | | |
